# Identification and Optimization of a Novel Taxanes Extraction Process from *Taxus cuspidata* Needles by High-Intensity Pulsed Electric Field

**DOI:** 10.3390/molecules27093010

**Published:** 2022-05-07

**Authors:** Zirui Zhao, Yajing Zhang, Huiwen Meng, Wenlong Li, Shujie Wang

**Affiliations:** College of Biology and Agricultural Engineering, Jilin University, Changchun 130022, China; zzirui94@163.com (Z.Z.); yajing20@mails.jlu.edu.cn (Y.Z.); 13834838037@163.com (H.M.); liwl21@mails.jlu.edu.cn (W.L.)

**Keywords:** *Taxus cuspidate* needles, taxanes, high-intensity pulsed electric field, response surface methodology, BP neural network–genetic algorithm, optimization

## Abstract

Taxanes are a series of natural compounds with great application potential in antitumor therapy, whereas the lack of efficient taxanes extraction methods significantly hinders the development of taxanes. The high-intensity pulsed electric field (PEF) is a novel technology used to extract bioactive ingredients from food and other natural products. However, the prospect of using PEF for taxanes extraction remains to be elucidated. Herein, we extracted taxanes from *Taxus cuspidata* via PEF and explored the effects of seven extraction conditions on the yields of target compounds. The Placket–Burman design (PBD) assay revealed that electric field strength, pulse number, and particle size are key factors for taxanes extraction. The response surface methodology (RSM) and back-propagation neural network conjugated with genetic algorithm (GA-BP) were further used to model and predict the optimal extraction conditions, and GA-BP exerted higher reliability, leading to a maximum extraction yield of 672.13 μg/g under electric field strength of 16 kV/cm, pulse number of 8, particle size of 160 meshes, solid–liquid ratio of 1:60, a single extraction, centrifugal speed of 8000 r/min, and flow rate of 7 mL/min, which was 1.07–1.84 folds that of control, solid–liquid extraction (SL), and ultrasonic extraction (US) groups. Additionally, the scanning electron microscopy (SEM) results indicated that the sample particles extracted by PEF method exhibited a coarser surface morphology. Thus, we present for the first time that PEF is feasible for the extraction of taxanes from *Taxus cuspidata* and highlight the application value of the PBD, RSM, and GA-BP models in parameters optimization during extraction process.

## 1. Introduction

**Figure 1:**
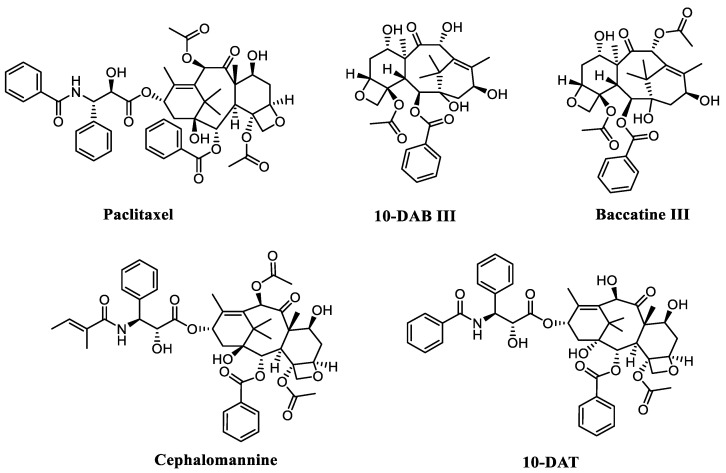
Chemical structures of five taxanes.

*Taxus cuspidata*, known as yew, is a small coniferous tree or shrub of *Taxaceae* and has great ornamental and medicinal values [1]. It has attracted considerable interest owing to its high contents of diterpene alkaloids, particularly paclitaxel and its precursors [2]. These compounds have a wide range of pharmacological activities and are widely employed to treat cancer patients owing to their potent antitumoral activity [3,4]. However, there are many difficulties in taxanes extraction that seriously hinder the large-scale production of target compounds. Presently, the most common procedure to obtain paclitaxel for clinical use is semi-synthesis from the precursor 10-deacetylbaccatins III (10-DAB III) [5]. Besides paclitaxel, the main method for obtaining its precursors, including 10-deacetylbaccatin III (10-DAB III), baccatine III, cephalomannine, and 10-deacetyltaxol (10-DAT) (Figure 1), is direct extraction from the bark, needles, and branches of Taxus plants via solid–liquid (SL) and ultrasonic extraction (US) technology, which have the shortcomings of high costs, low extraction rate, and long extraction time [6,7,8,9]. Therefore, it is urgent to find more novel and efficient taxanes extraction processes [10].

High-intensity pulsed electric field (PEF) is an emerging and highly concerning extraction method with low energy consumption and short treatment time [11]. Recently, PEF has been successfully used to extract bioactive components from different plants, foods, and by-product resources [9,12,13,14]. Specifically, PEF treatment effectively improves permeability of the cytoplasmatic membrane, thereby facilitating the release of the bioactive compounds from protoplasm [15]. An investigation into polyphenols extraction from orange peel revealed the potential of PEF as a gentle method to improve extraction efficiency and enhance the extracts’ antioxidant capacity [16]. Another study demonstrated that the combination of PEF and subcritical water extraction technology enhances the extraction efficiency of flavonoids from *C. unshiu* peel [9]. However, for *Taxus cuspidata*, there is no report on whether PEF is feasible for taxanes extraction, and the key factors that affect the extraction efficiency of this method remain to be elucidated. Additionally, considering that various conditions, including electric field strength, number of extractions, solid–liquid ratio, pulse number, centrifugal speed, flow rate, and particle size, have an impact on extraction effect, process optimization is also necessary to maximize the extraction efficiency [9]. Recently, response surface methodology (RSM) and artificial neural networks (ANN) have been employed in optimization of various processes synchronously or separately [13,17,18,19,20,21,22]. RSM, a compilation tool for mathematics and statistics widely used in engineering fields, is very useful for optimizing processes, especially when there are interactive effects between independent variables and response values [23,24]. Furthermore, RSM can predict the best experimental conditions according to a set of experimental results [25,26]. ANN is also fairly popular in constructing and optimizing processes, especially when the experimental values are limited [27,28,29]. By learning the relationship between observed values and created values, ANN can successfully create and construct complex nonlinear models to predict the best parameters [30,31]. Among various methods, the preferred method to improve the estimation accuracy of ANN models is the back-propagation (BP) algorithm [19,32]. Notably, many scholars successfully combine ANN and genetic algorithms (GA) to model and optimize the processing conditions [10,20,31,32,33].

In the present study, we aim to investigate the application potential of PEF to extract taxanes and characterize the optimal conditions to maximize the extraction efficiency. Our data show that PEF treatment effectively extracts taxanes from *Taxus Cuspidata.* Process optimization investigations using Plackett–Burman design (PBD), Box–Behnken design (BBD), RSM model, GA-BP model, and scanning electron microscopy (SEM) analysis were further performed to visually verify reliability of PEF treatment. Collectively, these findings highlight the potential of PEF as a novel method for taxanes extraction from *Taxus Cuspidata*.

## 2. Results and Discussion

### 2.1. Single-Factor Experiments

#### 2.1.1. Electric Field Strength

**Figure 2:**
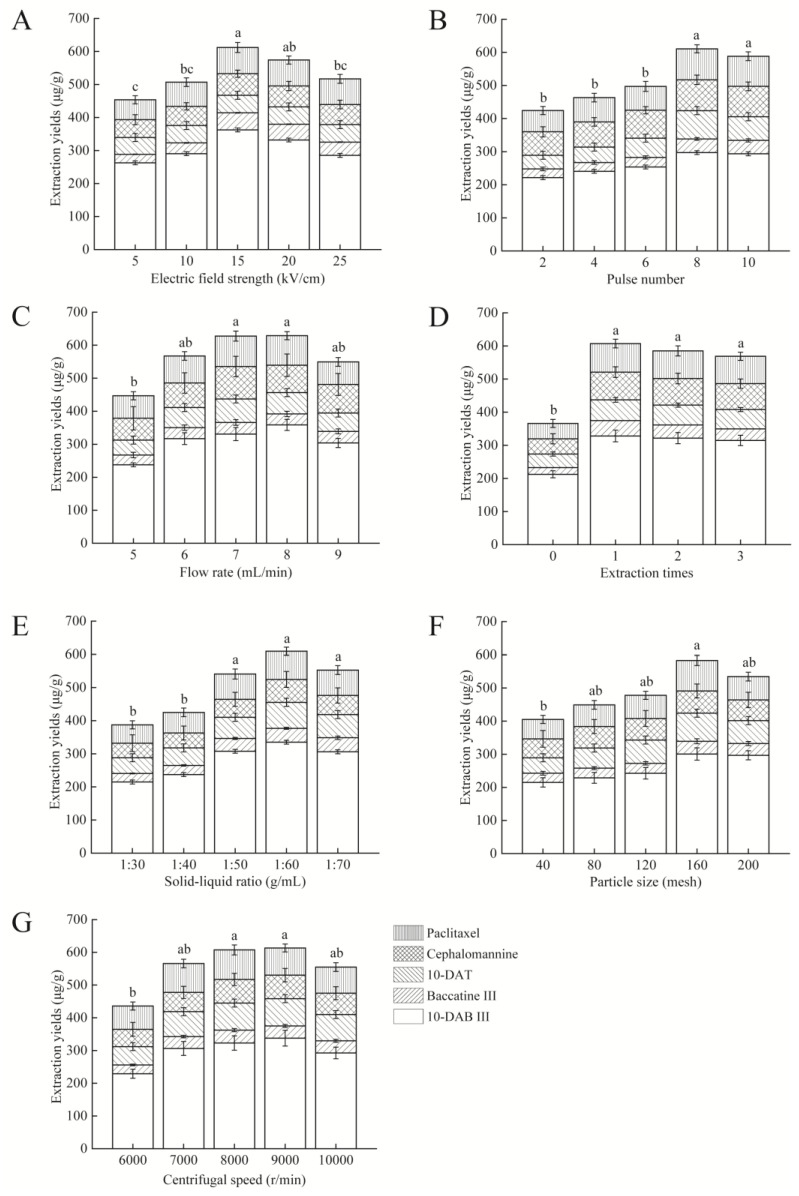
Effects of electric field strength (**A**), (**B**) pulse number, (**C**) flow rate, (**D**) number of extractions, (**E**) solid–liquid ratios, (**F**) particle size, and (**G**) centrifugation speed on extraction yields of five main taxanes in *Taxus cuspidata*. All data are presented as mean ± standard deviation. Data were collected from three independent experiments and analyzed using one-way analysis of variance. Different letters (a, b, c) in the same figure indicate significant differences (*p* < 0.05).

In the present study, we firstly investigate whether PEF is feasible for the extraction of taxanes. To this end, we first chose and explored the effects of electric field strength (from 5 to 25 kV/cm) on the extraction yields of five main taxanes from *Taxus cuspidata*. As shown in Figure 2A, with the increase in electric field strength ranging from 5 to 15 kV/cm, the contents of taxanes gradually increased and were maximized at 15 kV/cm, which are consistent with previous study [12]. These results can be explained by more complete cell lysis or the easier dissolution of taxanes under higher electric field strength. It is worth mentioning that the yields of the target compounds decreased when higher electric field strength was used. We speculate that this result may be associated with the following factors. As reported by Wang et al., the content of total taxanes in the extraction products decreased with raising temperature. It is reasonable to infer that taxanes may be degraded by the joule heating effect due to the thermal sensitivity of taxanes [34,35] or the volatilization of extraction solvents [36], or that taxanes may be reacted with the by-products (such as H_2_O_2_, hydrochloric acid, and hypochlorous acid) generated by the electrochemical reaction [37]. These speculations will be further explored in the following study.

In addition, irreversible damage to the electrodes occurs when excessive operating electric voltage is often used. We summarize the following points according to other scholars’ research [12,36,37,38,39,40]. First, the types and quantities of extracted compounds were expanded in the process. Some non-target products with a high degree of ionization in ethanol and dichloromethane were extracted, thereby increasing the conductivity and the current of solution. At the same time, with constant load on the sample being processed, excessive electric voltage will generate excessive electric current, leading to an increase in the processing temperature of the chamber. Second, the intensity of the electrochemical reactions in the processing chamber increases accordingly. Long-term continuous testing could lead to problems such as bubble breakdown (vaporization of ethanol and dichloromethane) and partial discharge-induced breakdown in the processing chamber, causing irreversible damage to the electrodes. Furthermore, under the high electrical parameters of the PEF, the metal ions released due to the corroded electrodes in the process also exacerbated the damage effect. Therefore, the extraction rate was dropped, and the electrode was damaged. Hence, 15 kV/cm was chosen for further research.

#### 2.1.2. Pulse Number

Pulse number is also a key factor affecting the extraction yield of taxanes. The data in Figure 2B show that as the pulse number gradually increased to 8, the yield of taxanes rose significantly (*p* < 0.05), but it did not increase further at 10 (*p* > 0.05). Increasing pulse number can lead to high-speed ions movement and intensify their collision with *Taxus cuspidata* [12]. However, the excessive number of pulses may aggravate the degradation of products, especially when treating with compounds with complex structures, which is proven in other studies [15]. The specific degradation mechanism and quantity will be investigated in our next study. Therefore, a pulse number of 8 was selected for subsequent experiments.

#### 2.1.3. Flow Rate

Flow rate, a significant characteristic parameter of PEF assay, reflects the residence time or extraction time inside the treatment zone [41]. Specifically, a faster flow rate is associated with a shorter extraction time and vice versa. Prolonged electrochemical reactions not only increase energy consumption but may also have an impact on the structure of taxanes. In the present study, we found that the extraction yields raised significantly with the increased flow rate and were maximized at 7 and 8 mL/min, although the extraction efficiency decreased at higher flow rate, possibly owing to incomplete dissociation of cells (Figure 2C). A similar trend was reported by Carbone et al. who extracted phenols from kiwi juice pomace [30]. Therefore, we conclude that a maximum speed of 7 mL/min is optimal.

#### 2.1.4. Number of Extractions

Conventional solvent extraction methods require repeated extraction to raise yields of target compounds [6]. To investigate whether PEF processing also requires multiple extractions, we explored the impact of number of extractions (0, 1, 2, and 3 times) on taxanes yields from *Taxus cuspidate*. As shown in Figure 2D, when the number of extractions was 1, the extraction yield of taxanes reached the highest, with a value of 607.27 μg/g. Interestingly, we observed that the extraction yields decreased when more extractions were performed, and we speculated that when continuous extraction experiments were carried out, the temperature of the reaction system may increase, thereby leading to degradation or reaction of target compounds due to thermal effects or excessive electrochemical reaction [14]. The exploratory research will be performed in the next study. Thus, the number of extractions 1 was selected for further experiments.

#### 2.1.5. Solid–Liquid Ratio

As the solid–liquid ratio in PEF assay is a major factor affecting extraction efficacy, we evaluated the effects of the solid–liquid ratio on the yield of taxanes further [42]. As shown in Figure 2E, with the rise in the liquid phase ratio, the extraction yield increased significantly (*p* < 0.05) and maximized at the ratio of 1:60, which could be explained by increasing the osmotic pressure difference inside and outside cells, leading to the accelerated diffusion of analytes [23]. These results are in accordance with other reports [42]. However, the extraction yields decreased with further increase in solvents. We speculated that the effect and energy of PEF were dispersed among liquids, thereby affecting the effective treatment of solid cells [12]. To sum up, for extracting taxanes by PEF, the solid–liquid ratio of 1:60 has highest extraction efficiency. Our findings are consistent with Wang et al., who reported that the recovery of BA was maximized at the liquid-to-material ratio of 20:1 and then steadily declined [43].

#### 2.1.6. Particle Size

It has been reported that samples with uniform particles size have better extraction effect [44]. In the present study, the extraction yield increased with the rise in grinding meshes until 160 meshes (*p* < 0.05) (Figure 2F). This may be because the larger meshes increase the surface area of particles, thereby enlarging the contact area with the solvent. Similar results were obtained in the studies on lemon peels [44] and *Psoralea Fructus* [45]. However, the reduced extraction yields were accompanied by the increase in particle size (from 160 to 200 meshes). These results accord with Chouaibi et al., who showed that the oil yield of *Citrullus colocynthis* L. seeds decreased as the particle size rose from 0.5 to 1 mm [28]. Suryawanshi and Bikash also uncovered a similar phenomenon in the seed oil extraction [29]. Therefore, 160 meshes were chosen for subsequent experiments.

#### 2.1.7. Centrifugal Speed

After PEF treatment, in order to obtain taxanes from the mixture of *Taxus cuspidate* powder and extractant and determine the extraction efficacy, we adopted the centrifugation method with a speed ranging from 6000 to 10,000 r/min to separate the target products. As shown in Figure 2G, the extraction yield of taxanes was highest at 8000 r/min, and there is no significant difference between 9000 and 10,000 r/min (*p* > 0.05). As for this difference, we speculate that the low centrifugal speed may not do well in achieving solid–liquid separation, thereby affecting the separation and collection of supernatants. Meanwhile, considering that excessive speed may lead to dispensable energy consumption, we therefore chose 8000 r/min for the next study.

### 2.2. Screening of Significant Factors Using PBD

PBD is an experimental design method used to screen a small number of important variables from multiple factors. Herewith, it was employed to screen the key factors of the PEF process for taxanes extraction. In this study, seven parameters (*A*: electric field strength, *B*: number of extractions, *C*: solid–liquid ratio, *D*: pulse number, *E*: centrifugal speed, *F*: flow rate, *G*: particle size) were selected as independent variables for further optimization. The factors and results of PBD assay are shown in Table 1. The ANOVA and regression analysis of PBD for prediction of significant extraction variables are presented in Table S1 from Supplementary Materials. According to ANOVA analysis (Table S1), this model does well in matching with the experimental results. Importantly, we can conclude that electric field strength (*A*), followed by pulse number (*D*) and particle size (*G*), are the key factors that affect taxanes extraction efficacy using PEF technology.

### 2.3. Construction of RSM Model and Conditions Optimization

Based on the PBD results, BBD was employed for further optimization to maximize the TEF yield (*Y*, the response value) by using electric field strength (*X*_1_), number of pulses (*X*_2_), and particle size (*X*_3_) as independent variables. The number of extractions, solid–liquid ratio, centrifugal speed, and flow rate were set as 1, 1:60, 8000 r/min, and 7 mL/min, respectively. The detailed experimental results of BBD assay are shown in Table 2, including the independent variables and predicted and actual values of TEF. The multiple quadratic regression equation is as shown below:

(1) $$Y=653.56+86.28X_{1}+47.53X_{2}+27.11X_{3}-37.42X_{1}X_{2}+6.65X_{1}X_{3}-52.48X_{2}X_{3}-81.71X_{1}^{2}-52.60X_{2}^{2}-75.16X_{3}^{2}$$

where *X*_1_, *X*_2_, and *X*_3_ denote the variables (coded) for the electric field strength (*X*_1_), number of pulses (*X*_2_), and particle size (*X*_3_), respectively, and *Y* is the TEF.

Based on above data, RSM model was built, and ANOVA was employed to evaluate the accuracy of the model. ANOVA for RSM is shown in Table S2 from Supplementary Materials. As shown in Table S2, it is evident that the RSM model could do well in predicting the optimum experimental conditions. Based on this, we further evaluated the interactive effects of three experimental factors on TEF extraction process.

**Figure 3:**
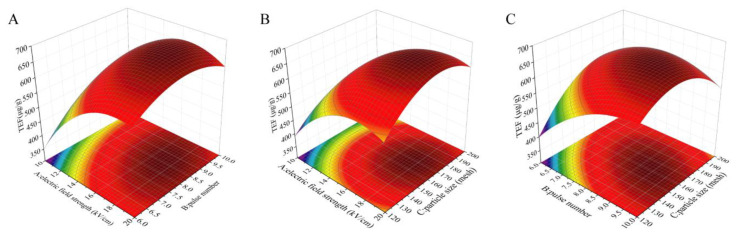
Interaction diagrams of various factors: (**A**) Electric field strength and pulse number; (**B**) Electric field strength and particle size; (**C**) Pulse number and particle size.

As shown in Figure 3, the results reveal that the mutual effect of pulse number (*X*_2_) and particle size (*X*_3_) is the strongest, while the mutual effect of electric field strength (*X*_1_) and particle size (*X*_3_) is the weakest. With an increase in the pulse number (*X*_2_) and particle size (*X*_3_), the extraction yield increased first and then reduced, which was in accordance with the single-factor results. Based on the multiple quadratic regression equation, we calculated the optimal experimental conditions of electric field strength, pulse number, and particle size, with the values of 17.419 kV/cm, 8.435, and 165.041 meshes, respectively. Under these conditions, we performed the taxanes extraction assay to evaluate the reliability of the RSM model. As shown in Table 3, the prediction and experimental values of the taxanes yields were 681.282 and 658.15 μg/g, respectively, and *p* value was 0.269. All data highlight that the RSM model is feasible for parameters optimization during taxanes extraction process.

### 2.4. Construction of GA-BP Modeling and Conditions Optimization

Owing to the advantages of low cost, timeliness, and high accuracy, ANN models are widely used in nonlinear system prediction [21]. BP, the most popular ANN model, was employed to model the taxanes contents. This network was constructed according to the RSM experimental and virtual data (Table S3). The virtual samples, the performance of the ANN model and regression plot of the ANN model are shown in Table S3, Figures S1 and S2 from Supplementary Materials, respectively. For the simulation results of the BP model, we observed that the mean squared error was lower than 0.01 (Figure S1) and the correlation coefficient was higher than 0.99 (Figure S2), which indicates that the trained ANN model has a superior predictive ability.

**Figure 4:**
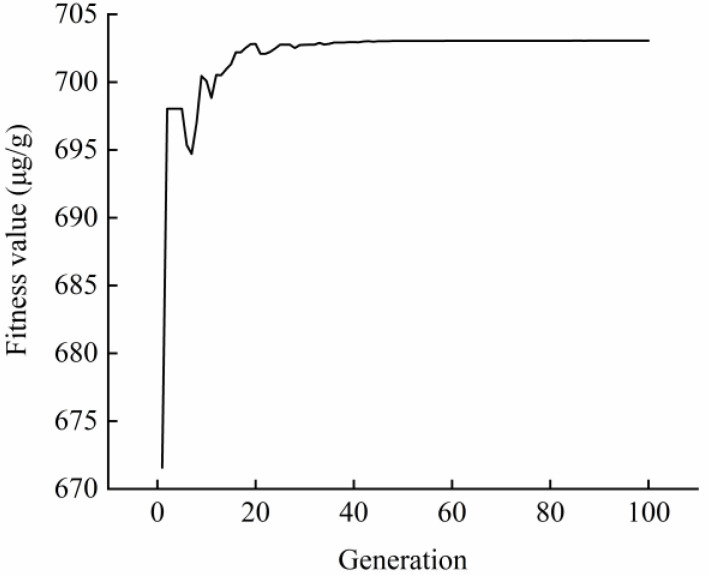
Fitness curve of GA-BP model.

Furthermore, we adopted the GA method to fit and optimize the BP model. As shown in Figure 4, the fitness value changed with the increase in generations. The linear weighted sum of each sub-objective became invariable after 44 generations, and the population gradually converged. To summarize, the above results suggest that we successfully constructed and optimized the GA-BP model for parameters optimization of taxanes extraction. Based on the optimum conditions predicted by this model, we also carried out the assay to compare the difference between prediction and experimental results, with the values of 703.0565 and 672.13 μg/g, respectively (Table 3).

In the present study, compared with the RSM model, the GA-BP model exhibited a better prediction performance (Table S4), which is in line with other scholars [10,24,30]. Evaluation of the predictive capacities of RSM and GA-BP modeling systems are shown in Table S4 from Supplementary Materials. Under the recommended conditions of RSM and GA-BP, the experimental TEFs were 658.15 ± 31.26 and 672.13 ± 32.45 μg/g, respectively. In addition, as shown in Table 3, it is evident that the prediction and optimization capabilities of RSM are not as good as those of GA-BP. We herein speculate that the higher TEF under the recommended process parameters of GA-BP may be attributed to the lower electric field strength as well as pulse number.

### 2.5. The Extraction Efficacy of PEF Compared with the Conventional Methods

**Figure 5:**
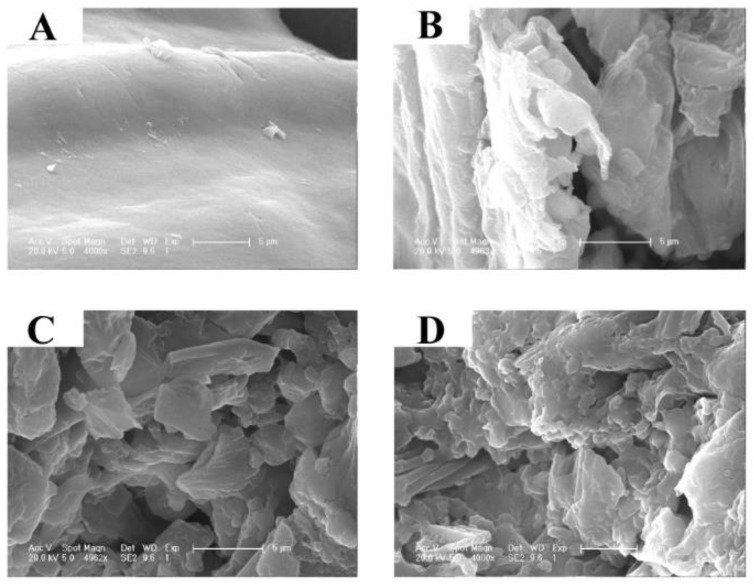
SEM images of the control (**A**), SL (**B**), US (**C**) and PEF (**D**) treated groups.

Until now, taxanes have been mainly extracted from *Taxus* needles via solid–liquid extraction method, ultrasonic extraction method, etc. [7]. To determine the difference in extraction efficacy between PEF technology and the conventional methods, we compared the yields of taxanes in different treatment groups, including the control group, the SL group, and the US group. All groups adopted the same solvents and solid–liquid ratio. As shown in Table 4, it was apparent that the yields of 10-DAB III, baccatine III, 10-DAT, cephalomannine, paclitaxel, and TEF by PEF treatment were 1.02–1.66, 1.24–2.80, 1.09–1.78, 1.24–2.20, 1.06–1.98, and 1.07–1.84 folds those of control, SL and US treatment, which indicates the extraction yields of PEF treatment were much higher than conventional treatments. Compared with the control group, SL group treated the samples with a higher temperature and more extractions, thereby leading to the increase in taxanes yields. However, the process of this method inevitably utilizes a large amount of organic solvent and takes a long time, which may cause the degradation and coagulation of active ingredients [46]. The yields of TEF in US group was significantly enhanced; however, there are some drawbacks for US treatment, such as the fact that prolonged ultrasonic treatment and its related thermal effect may destroy the functional structure of target compounds and accelerate the volatilization of the solvents. In the present study, we highlight the potential application value of PEF technology as a promising method for taxanes extraction, which exhibits a higher extraction efficacy and shorter time compared with the conventional methods. As the extraction solvent composed of dichloromethane and ethanol (1:1 *v*/*v*) used in this experiment is not a green reagent, we therefore emphasize here that the further optimization direction is to replace the organic solvents with clean, non-toxic, green, and highly specific extractants and use them in conjunction with PEF technology to maximize extraction yields. Furthermore, considering that the extraction rate is closely associated with the microstructure changes of particles, and in order to further verify the above results and visually compare the differences among every group, we performed SEM assays to observe the surface morphology of *Taxus cuspidate*. As shown in Figure 5, the samples of the control group presented a smooth and intact surface, whereas the surface of *Taxus cuspidata* in other treated groups, especially in PEF treated group, were rough with a few irregularly shaped cavities, which enlarge the surface area of particles and reduce the mass transfer resistance due to the irreversible electroporation process [14]. Collectively, these results all support the potential usage of PEF for taxanes extraction.

## 3. Materials and Methods

### 3.1. Materials and Reagents

The annual stems and leaves of *Taxus cuspidata* collected from Changbai Mountain (Jilin, China) were washed and dried at 40 °C to constant weights. Then, the samples were crushed and sealed for storage in a dry environment.

Dichloromethane and ethanol (both analytical-grade) were purchased from Damao Chemical Reagent Factory (Tianjin, China). Acetonitrile and methanol (both chromatographic grade) were provided by Thermo Fisher Scientific (Rockville, MD, USA). Purified water was produced by Hangzhou Wahaha Company (Hangzhou, China). Paclitaxel, 10-deacetylbaccatin III (10-DAB III), baccatine III, cephalomannine, and 10-deacetyltaxol (10-DAT) with purity >98% were bought from Shanghai Yuanye Bio-Technology Co., Ltd. (Shanghai, China).

### 3.2. PEF Treatment

The PEF extraction system used here involves a high-voltage pulse generator, an oscilloscope, a coaxial liquid material processing chamber, and a pump [12].

The generator was formed by two 1.5-mm-long stainless steel electrodes. The chamber diameter was set as 0.1 cm for processing samples. The bidirectional triangular pulse wave form with real-time voltage and current were displayed on the oscilloscope. The pulse width, frequency range, peak output voltage, and current were 2 μs, 40–3000 Hz, 10 kV, and 20 A, respectively. The electric field strength and pulse number were changed by adjusting the input voltage and frequency. The calculation formulas are as follows:

(2) $${n=\pi r}^{2}lf/10^{3}v$$

(3) $$E{=V}_{pp}/2l$$

where *n* is the pulse number, *r* is the electrode radius (0.5 mm), *l* is the distance between electrodes (1.5 mm), *f* is frequency (Hz), *v* is flow rate (mL/s), *E* is the electric field strength (kV/cm), and *V_pp_* is the displayed real-time voltage on the oscillograph (kV).

The PEF extraction assay was performed as described previously with some modifications [12]. Briefly, the dried *Taxus cuspidata* powder was firstly mixed with the extractant, which was a mixture of ethanol and dichloromethane (1:1, *v*/*v*). Then, the parameters, including the pulse number and flow rate of the PEF instrument, were set according to the experimental protocol. The peristaltic pump was switched on, and the treatment liquid consisting of *Taxus cuspidata* powder and extractant was pumped into the chamber. After the treatment liquid flowed through both electrodes stably and continuously, the voltage switch was turned on and set as required. Under this process, the taxanes were extracted from *Taxus cuspidata* powder into the extractant. Finally, the products were collected for centrifugation.

### 3.3. Experimental Design

#### 3.3.1. Single-Factor Experiment

Since the taxanes yield of *Taxus cuspidata* may be influenced by various factors, single-factor experiments were designed and performed. The experimental ranges of different factor used in this study were electric field strength = 5–25 kV/cm, number of extractions = 0–3, solid–liquid ratio = 1:30–1:70 g/mL, pulse number = 2–10, centrifugal speed = 6000–10,000 r/min, flow rate = 4–8 mL/min, and particle size = 40–200 meshes.

#### 3.3.2. PBD

To identify the key factors in PEF extraction assay, we performed PBD assay using Design-Expert 10 (Stat-Ease, Inc., Minneapolis, MN, USA) and narrowed the boundary of each variable according to the single-factor test results [45]. The specific boundaries are as follows: electric field strength (*A*) = 10–20 kV/cm, number of extractions (*B*) = 1–2, solid–liquid ratio (*C*) = 1:50–1:70 g/mL, pulse number (*D*) = 6–10, centrifugal speed (*E*) = 7000–9000 r/min, flow rate (*F*) = 6–8 mL/min, and particle size (*G*) = 120–200 meshes.

#### 3.3.3. RSM for Extraction Optimization

Among the design methods of RSM, the experiment involving the tangent point and center point of the cube and the sphere is BBD assay, which requires fewer tests in comparison with other statistical designs [25,43]. According to the results of PBD, electric field strength (*X*_1_), pulse number (*X*_2_), and particle size (*X*_3_) were deemed as the main independent variables, while TEF (*Y*) was selected as the dependent variable. A total of 17 groups of experiments were carried out. For statistical calculations, the independent variables were coded at three levels (−1, 0, 1) according to Xu et al. [25].

### 3.4. Detection of Taxanes

After PEF treatment, the suspension of the solution was centrifuged at 8000 r/min for 10 min at 4 °C, and then the dichloromethane-ethanol layer was gathered to evaporate at ≤40 °C until complete drying. After that, the residues were dissolved in methanol, passed through a 0.22 μm filter membrane, and stored in a brown bottle at −20 °C for further analysis.

Content of each type of taxanes in the extracts was quantified using a high-performance liquid chromatograph (HPLC, Waters e2695 Separations Module) according to Fan et al. [7] with some modifications. Taxanes were separated through a C18 reverse-phase column (5 μm 4.6 × 250 mm^2^). The flow rate, column temperature, and injection volume were set as 1.0 mL/min, 30 °C, and 10 μL, respectively. The mobile phase consisted of water (eluent A) and acetonitrile (eluent B). The gradient elution procedure was set as follows: 40 to 50% B (1–10 min), 50 to 53% B (10–13 min), 53 to 73% B (13–25 min), 73 to 40% B (25–27 min), and 40% B (27–30 min). The detection wavelength was 227 nm. Five main taxanes were identified by matching the spectral characteristics between the samples and standards. The concentrations of paclitaxel, 10-DAB III, baccatine III, cephalomannine and 10-DAT were calculated using the calibration curves. The total content of the five main taxanes (*TEF*, μg taxanes equivalents/g of *Taxus cuspidata*) was calculated as follows:

(4) TEF=C × V/W

where *C* (μg/mL) is the total content of five main taxanes, *V* (mL) is the volume of extraction solution recovered, *W* (g) is the weight of *Taxus cuspidata*, and *TEF* (μg/g) represents the extraction rate of taxanes in *Taxus cuspidata*.

### 3.5. Models and Optimization

#### 3.5.1. RSM Model

Response surface regression was used to analyze the experimental data of BBD.

The relationship between response and the independent variables was described by a second-order polynomial model [18]:

(5) $${Y=\beta }_{0}+\sum_{i=1}^{k}\beta_{i}X_{i}+\sum_{i=1}^{k}\beta_{ii}X_{ii}^{2}+\sum_{i}^{k-1}\sum_{j}^{k}\beta_{ij}X_{ij}$$

where *Y* was the dependent variables; *X_i_* and *X_j_* were independent coded variables; *β*_0_ was the constant term; *β_i_* was the linear effect; *β_ii_* was the quadratic effect; and *β_ij_* was the interaction effect.

Analysis of variance (ANOVA) was employed to evaluate the correctness of the polynomial model and find out the significant influence factors on TEF [21]. Regression analysis and three-dimensional RSM were used to determine the optimal extraction conditions.

#### 3.5.2. ANN Conjugated with Genetic Algorithm (GA-BP) Model

The taxanes extraction process from *Taxus cuspidate* was modeled using a feed-forward BP learning algorithm and realized via MATLAB (R2020b) (Mathworks Inc., Natick, MA, USA). A three-layer ANN structure composed of an input layer, a hidden layer, and an output layer was generated [24]. The three input factors, including electric field strength, pulse number, and particle size, were normalized using the mapminmax function to obtain more accurate model results. TEF was taken as the output. The transfer functions of the hidden layer and output layer were hyperbolic tangent sigmoid (tansig) and linear (purelin), respectively [20,32,42]. To avoid over-fitting, we only set one hidden layer in the model constructed here [20]. The neuron number of the hidden layer was determined with minimum mean square error (MSE) and maximum R-value of neural network topology based on training, validation, and testing [27]. Finally, ten neurons were set in the hidden layer (Figure S3). ANN structure of PEF extraction of five main taxanes from *Taxus cuspidate* were presented in Figure S3 from Supplementary Materials.

Based on the experiment errors of independent variables and large bodies of ANN, virtual samples were set to improve the self-studying and self-adjusting abilities. Virtual samples were generated by adding ±Δ*i* (Δ*i* = 0.2%) to each variable of an actual sample, according to Wen et al. [47,48]. Eight virtual samples were generated for each actual sample (Table S3). The virtual samples are shown in Table S3 from Supplementary Materials. In total, 153 samples were acquired with the addition of 17 actual samples to establish the ANN model and predict the TEF. About 70, 15, and 15% of the overall experimental datasets were utilized for training, validation, and testing of the ANN model, respectively, according to Inyang et al. with some modifications [22].

Then, GA was used to optimize the inputs (independent variables) of the ANN model to maximize the TEF yield. The parameters used in GA optimization were the same as those described by Abdullah et al., except for the cross score, which was set to be 0.3 [32]. The optimal solution was outputted after repeated selection, crossover, and mutation until the population converged [10]. To validate and compare the performance of RSM and GA-BP models, we calculated statistical measures according to Dey et al. [27].

### 3.6. Verification and Comparsion of the Optimized Extraction Conditions

After the optimization of taxanes extraction conditions, three independent experiments were carried out to evaluate the difference between the experimental values and the predicted values, so as to determine the superiority of the prediction model.

To compare the extraction efficacy of TEF and the conventional extraction methods, four treatment groups, including the control group, the SL group, the US group, and the PEF group, were set to extract the taxanes. For the control group, the experiment was performed by using a dichloromethane-ethanol system without PEF treatment. For SL treatment group, 2 g *Taxus cuspidata* powder was accurately weighed and extracted with 40 mL of dichloromethane-ethanol (1:1, *v*/*v*) solution in a reflux device at 40 °C. After 4 h, the suspension was centrifugated to collect the supernatants. The precipitate was extracted twice under the above conditions. Finally, a total of 120 mL suspension was used to determine the taxanes content. For US treatment group, the experiment was conducted as described previously with some modifications [6]. Briefly, 2 g of *Taxus cuspidata* powder was mixed with 120 mL of extractant and placed in a sonication chamber. The extractant was a 1:1 mixture of dichloromethane and ethanol. The temperature, power, and duration of ultrasonic treatment were set as 40 °C, 200 W, and 1.1 h, respectively.

### 3.7. SEM

The microstructures of residues after control, SL, US, and PEF treatments were observed by an XL-30 ESEM-FEG SEM device (FEI Company, Hillsboro, OR, USA). Before observation, all samples were processed as described by Xu et al. [25].

### 3.8. Method of Analysis

All experiments were conducted three times, and the results were expressed as mean ± standard deviation. Significant differences were set at *p* < 0.05 by ANOVA, Duncan’s new multiple range test, or *t*-test using IBM SPSS Statistics 25.0 (IBM, New York, NY, USA).

## 4. Conclusions

In the present study, we showed that PEF is an effective and eco-friendly method for extracting taxanes from *Taxus cuspidata* needles. The maximum taxanes yields was obtained at 16 kV/cm of electric field strength, pulse number of 8, and particle size of 160 meshes of particle size, which were 1.09–1.72 and 1.02–1.24 folds in fewer extractions to those by the conventional SL and US methods, respectively. In addition, RSM model and GA-BP model could be employed to determine the optimum conditions of PEF treatment. To sum up, these findings highlight the potential of PEF as a novel method to extract hydrophobic bioactive ingredients from other plant materials and also provides an effective method for comprehensively exploring the effect of process parameters on the results.

## Figures and Tables

**Table 1:** Factors and results of PBD.

No.	*A* (kV/cm)	*B* (time)	*C* (g/mL)	*D*	*E* (r/min)	*F* (mL/min)	*G* (mesh)	TEF (μg/g)
1	1 (20)	−1 (1)	−1 (1:50)	−1 (6)	1 (9000)	−1 (6)	1 (200)	620.00 ± 38.24
2	−1 (10)	−1	−1	−1	−1 (7000)	−1	−1 (120)	516.67 ± 29.82
3	−1	−1	−1	1 (10)	−1	1 (8)	1	496.64 ± 31.63
4	−1	1 (2)	−1	1	1	−1	1	483.35 ± 30.73
5	1	1	1 (1:70)	−1	−1	−1	1	623.23 ± 36.55
6	1	1	−1	1	1	1	−1	556.58 ± 32.72
7	−1	1	1	−1	1	1	1	543.71 ± 35.95
8	1	−1	1	1	1	−1	−1	540.00 ± 30.45
9	−1	−1	1	−1	1	1	−1	513.16 ± 36.84
10	1	1	−1	−1	−1	1	−1	580.00 ± 37.16
11	1	−1	1	1	−1	1	1	576.78 ± 34.57
12	−1	1	1	1	−1	−1	−1	490.00 ± 21.28

*A*: electric field strength (kV/cm), *B*: number of extractions, *C*: solid–liquid ratio (g/mL), *D*: pulse number, *E*: centrifugal speed (r/min), *F*: flow rate (mL/min), *G*: particle size (mesh), TEF: taxanes equivalents of *Taxus cuspidate* (μg/g).

**Table 2:** Experimental design and results of BBD.

Order	*X*_1_	*X*_2_	*X*_3_	TEF/(μg/g)		
				Experimental	RSM Predicted	ANN Predicted
1	0 (15)	1 (10)	−1 (120)	613.23 ± 38.24	598.70	613.18
2	−1 (10)	−1 (6)	0 (160)	366.68 ± 27.63	348.03	366.72
3	−1	1	0	513.35 ± 35.94	517.92	512.91
4	0	0 (8)	0	644.00 ± 49.35	653.56	651.70
5	0	0	0	663.52 ± 56.26	653.56	651.70
6	1 (20)	0	−1	553.34 ± 41.54	549.22	553.41
7	0	−1	1 (200)	543.35 ± 39.64	557.88	542.90
8	0	−1	−1	390.00 ± 29.53	398.69	389.96
9	−1	0	1	426.76 ± 32.63	430.88	427.06
10	0	0	0	673.61 ± 56.56	653.56	651.70
11	0	0	0	626.68 ± 47.85	653.56	651.70
12	−1	0	−1	380.00 ± 22.92	389.96	380.08
13	0	0	0	660.00 ± 54.36	653.56	651.70
14	1	1	0	597.00 ± 38.74	615.65	596.97
15	0	1	1	556.65 ± 39.24	547.96	557.50
16	1	−1	0	600.00 ± 42.94	595.43	600.29
17	1	0	1	626.70 ± 43.86	616.74	626.62

*X*_1_: electric field strength (kV/cm), *X*_2_: number of pulses, *X*_3_: particle size (mesh), TEF: taxanes equivalents of *Taxus cuspidate* (μg/g), RSM: response surface methodology, ANN: artificial neural network.

**Table 3:** Validation optimized conditions of RSM and GABP model.

Methods	Optimum Values			TEF (μg/g)		
	*X*_1_	*X*_2_	*X*_3_	Predicted	Experimental	*p*-Value
RSM	17.419	8.435	165.041	681.282	658.15 ± 31.26	0.269
GA-BP	15.5265	8.2551	165.1023	703.0565	672.13 ± 32.45	0.174

*X*_1_: electric field strength (kV/cm), *X*_2_: pulse number, *X*_3_: particle size (mesh), RSM: response surface methodology, GA-BP: back-propagation neural network conjugated with genetic algorithm, TEF: taxanes equivalents of *Taxus cuspidate* (μg/g), *p*-values < 0.05 indicates significance.

**Table 4:** The content of five compounds extracted with different methods.

Methods	Yields (μg/g)					
	10-DAB III	Baccatine III	10-DAT	Cephalomannine	Paclitaxel	TEF
Control	212.39 ± 10.71	20.22 ± 1.83	50.99 ± 6.09	35.96 ± 15.28	46.39 ± 12.39	365.95 ± 19.68
SL	264.05 ± 15.97	36.83 ± 2.04	57.30 ± 12.45	46.02 ± 15.41	85.11 ± 12.83	489.31 ± 21.47
US	347.63 ± 17.81	45.66 ± 2.25	83.28 ± 7.11	63.94 ± 16.02	86.21 ± 13.10	626.72 ± 25.63
PEF	353.85 ± 18.62	56.62 ± 7.51	90.92 ± 12.24	78.97 ± 21.02	91.77 ± 15.22	672.13 ± 32.45

Control: untreated sample, SL: solid–liquid extraction, US: ultrasonic extraction, PEF: high-intensity pulsed electric field, TEF: taxanes equivalents of *Taxus cuspidate* (μg/g), 10-DAB III: 10-deacetylbaccatin III, 10-DAT: 10-deacetyltaxol.

## Data Availability

The authors declare that all the data supporting the findings of this study are available within the article or supplementary material.

## Supplementary Materials

The following supporting information can be downloaded at: https://www.mdpi.com/article/10.3390/molecules27093010/s1, Table S1: ANOVA and regression analysis of PBD for prediction of significant extraction variables; Table S2: ANOVA for response surface polynomial model; Table S3: Virtual samples; Figure S1: The performance of the ANN model; Figure S2: Regression of the ANN model for training, testing, validation, and all data sets; Table S4: Evaluation of the predictive capacities of RSM and GA-BP modeling systems; Figure S3: ANN structure of PEF extraction of five main taxanes from *Taxus cuspidate*.

## Sample Availability

Samples of the compounds are available from the corresponding authors.

## References

[1] Shao F., Wilson I.W., Qiu D. (2020). The Research Progress of taxol in Taxus. Curr. Pharm. Biotechnol..

[2] Malik S., Cusidó R.M., Mirjalili M.H., Moyano E., Palazón J., Bonfill M. (2011). Production of the anticancer drug taxol in *Taxus baccata* suspension cultures: A review. Process Biochem..

[3] Gallego-Jara J., Lozano-Terol G., Sola-Martinez R.A., Canovas-Diaz M., de Diego Puente T. (2020). A Compressive Review about Taxol^®^: History and Future Challenges. Molecules.

[4] Cusido R.M., Onrubia M., Sabater-Jara A.B., Moyano E., Bonfill M., Goossens A., Angeles Pedreno M., Palazon J. (2014). A rational approach to improving the biotechnological production of taxanes in plant cell cultures of *Taxus* spp.. Biotechnol. Adv..

[5] Miele M., Mumot A.M., Zappa A., Romano P., Ottaggio L. (2012). Hazel and other sources of paclitaxel and related compounds. Phytochem. Rev..

[6] Wang S.J., Li C., Wang H.J., Zhong X.M., Zhao J., Zhou Y.J. (2016). A process optimization study on ultrasonic extraction of paclitaxel from *Taxus cuspidata*. Prep. Biochem. Biotechnol..

[7] Fan X., Wang L., Chang Y., An J., Zhu Y., Yang Q., Meng D., Fu Y. (2021). Application of green and recyclable menthol-based hydrophobic deep eutectic solvents aqueous for the extraction of main taxanes from *Taxus chinensis* needles. J. Mol. Liq..

[8] Kim G., Kim J. (2015). Enhancement of extraction efficiency of paclitaxel from biomass using ionic liquid-methanol co-solvents under acidic conditions. Process Biochem..

[9] Hwang H.J., Kim H.J., Ko M.J., Chung M.S. (2021). Recovery of hesperidin and narirutin from waste *Citrus unshiu* peel using subcritical water extraction aided by pulsed electric field treatment. Food Sci. Biotechnol..

[10] Yu Z., Zhang Y., Zhao X., Yu L., Chen X., Wan H., He Y., Jin W. (2021). Simultaneous optimization of ultrasonic-assisted extraction of Danshen for maximal tanshinone IIA and salvianolic acid B yields and antioxidant activity: A comparative study of the response surface methodology and artificial neural network. Ind. Crop. Prod..

[11] Yamakage K., Yamada T., Takahashi K., Takaki K., Komuro M., Sasaki K., Aoki H., Kamagata J., Koide S., Orikasa T. (2021). Impact of pre-treatment with pulsed electric field on drying rate and changes in spinach quality during hot air drying. Innov. Food Sci. Emerg..

[12] He G., Yan X., Wang X., Wang Y. (2019). Extraction and structural characterization of collagen from fishbone by high intensity pulsed electric fields. J. Food Process Eng..

[13] He G., Yin Y., Yan X., Wang Y. (2017). Semi-Bionic Extraction of Effective Ingredient from Fishbone by High Intensity Pulsed Electric Fields. J. Food Process Eng..

[14] Ranjha M., Kanwal R., Shafique B., Arshad R.N., Irfan S., Kieliszek M., Kowalczewski P.L., Irfan M., Khalid M.Z., Roobab U. (2021). A Critical Review on Pulsed Electric Field: A Novel Technology for the Extraction of Phytoconstituents. Molecules.

[15] Tzima K., Brunton N.P., Lyng J.G., Frontuto D., Rai D.K. (2021). The effect of Pulsed Electric Field as a pre-treatment step in Ultrasound Assisted Extraction of phenolic compounds from fresh rosemary and thyme by-products. Innov. Food Sci. Emerg..

[16] Luengo E., Álvarez I., Raso J. (2013). Improving the pressing extraction of polyphenols of orange peel by pulsed electric fields. Innov. Food Sci. Emerg..

[17] Bi Y., Chi X., Zhang R., Lu Y., Wang Z., Dong Q., Ding C., Yang R., Jiang L. (2020). Highly efficient extraction of mulberry anthocyanins in deep eutectic solvents: Insights of degradation kinetics and stability evaluation. Innov. Food Sci. Emerg..

[18] Panda G., Vivek K., Mishra S., Pradhan R.C. (2021). Characterization and Optimization of Process Parameters for Enzyme Assisted Extraction of Kendu (*Diospyros Melanoxylon* Roxb.) Fruit Juice. Int. J. Fruit Sci..

[19] Uslu S. (2020). Optimization of diesel engine operating parameters fueled with palm oil-diesel blend: Comparative evaluation between response surface methodology (RSM) and artificial neural network (ANN). Fuel.

[20] Pradhan D., Abdullah S., Pradhan R.C. (2020). Optimization of Pectinase Assisted Extraction of Chironji (*Buchanania Lanzan*) Fruit Juice Using Response Surface Methodology and Artificial Neural Network. Int. J. Fruit Sci..

[21] Zhao Z., Sun W., Ray M.B., Ray A.K., Huang T., Chen J. (2019). Optimization and modeling of coagulation-flocculation to remove algae and organic matter from surface water by response surface methodology. Front. Env. Sci. Eng..

[22] Inyang V., Lokhat D. (2022). Propionic acid recovery from dilute aqueous solution by emulsion liquid membrane (ELM) technique: Optimization using response surface methodology (RSM) and artificial neural network (ANN) experimental design. Sep. Sci. Technol..

[23] Bianchin M., de Lima H.H.C., Monteiro A.M., Benassi M.d.T. (2020). Optimization of ultrasonic-assisted extraction of kahweol and cafestol from roasted coffee using response surface methodology. LWT.

[24] Yang Q., Gan R., Zhang D., Ge Y., Cheng L., Corke H. (2019). Optimization of kidney bean antioxidants using RSM & ANN and characterization of antioxidant profile by UPLC-QTOF-MS. LWT.

[25] Xu N., Sun Y.H., Guo X.L., Liu C., Mao Q., Hou J.M. (2017). Optimization of ultrasonic-microwave synergistic extraction of polysaccharides from *Morchella conica*. J. Food Process. Pres..

[26] Kim M., Nam D.G., Choe J.S., Hwang K.A., Choi A.J. (2021). Optimization of pectinase-assisted extraction condition of mulberry (*Morus alba* L.) fruit using response surface methodology and its effect on anthocyanin synthesis pathway-related metabolites. J. Food Sci..

[27] Dey S., Reang N.M., Das P.K., Deb M. (2021). Comparative study using RSM and ANN modelling for performance-emission prediction of CI engine fuelled with bio-diesohol blends: A fuzzy optimization approach. Fuel.

[28] Chouaibi M., Rigane K., Ferrari G. (2020). Extraction of *Citrullus colocynthis* L. seed oil by supercritical carbon dioxide process using response surface methodology (RSM) and artificial neural network (ANN) approaches. Ind. Crop. Prod..

[29] Suryawanshi B., Mohanty B. (2018). Application of an artificial neural network model for the supercritical fluid extraction of seed oil from *Argemone mexicana* (L.) seeds. Ind. Crop. Prod..

[30] Carbone K., Amoriello T., Iadecola R. (2020). Exploitation of Kiwi Juice Pomace for the Recovery of Natural Antioxidants through Microwave-Assisted Extraction. Agriculture.

[31] Pradhan D., Abdullah S., Pradhan R.C. (2021). Chironji (*Buchanania lanzan*) fruit juice extraction using cellulase enzyme: Modelling and optimization of process by artificial neural network and response surface methodology. J. Food Sci. Technol..

[32] Abdullah S., Pradhan R.C., Aflah M., Mishra S. (2020). Efficiency of tannase enzyme for degradation of tannin from cashew apple juice: Modeling and optimization of process using artificial neural network and response surface methodology. J. Food Process Eng..

[33] Khawas P., Dash K.K., Das A.J., Deka S.C. (2015). Modeling and optimization of the process parameters in vacuum drying of culinary banana (*Musa* ABB) slices by application of artificial neural network and genetic algorithm. Drying Technol..

[34] Paoli M., Bighelli A., Castola V., Tomi F., Casanova J. (2013). Quantification of taxanes in a leaf and twig extract from *Taxus baccata* L. using ^13^C-NMR spectroscopy. Magn. Reson. Chem..

[35] Wang Y., Gamage J., Zhang Z. (2011). Separation of taxanes from *Taxus canadensis* using dynamic pressurized liquid extraction. Biotechnol. Biopro. E..

[36] Ruobing Z., Nanchen Z., Huaiyu L., Liming W. (2015). Influencing Factors of Dielectric Breakdown in the PEF Treatment Chamber. IEEE T. Plasma Sci..

[37] Jayaram S.H. (2000). Sterilization of liquid foods by pulsed electric fields. IEEE Electr. Insul. Mag..

[38] Lyu C. (2018). Prevention of Bubble Breakdown and Electrolysis in the Pulsed Electric Fields Treatment Chamber Assisted by Ultrasound and Microfluidics. Ph.D. Thesis.

[39] Pataro G., Falcone M., Donsì G., Ferrari G. (2014). Metal release from stainless steel electrodes of a PEF treatment chamber: Effects of electrical parameters and food composition. Innov. Food Sci. Emerg..

[40] Roodenburg B., Morren J., Berg H.E., de Haan S.W.H. (2005). Metal release in a stainless steel pulsed electric field (PEF) system. Innov. Food Sci. Emerg..

[41] Meneses N., Jaeger H., Moritz J., Knorr D. (2011). Impact of insulator shape, flow rate and electrical parameters on inactivation of *E. coli* using a continuous co-linear PEF system. Innov. Food Sci. Emerg. Technol..

[42] Portillo-Lopez R., Morales-Contreras B.E., Lozano-Guzman E., Basilio-Heredia J., Muy-Rangel M.D., Ochoa-Martinez L.A., Rosas-Flores W., Morales-Castro J. (2021). Vegetable oils as green solvents for carotenoid extraction from pumpkin (*Cucurbita argyrosperma* Huber) byproducts: Optimization of extraction parameters. J. Food Sci..

[43] Wang C.-Y., Yan C.-S., Cai J.-M. (2019). Optimization of Extraction Parameters for Blueberry Anthocyanins. Curr. Top. Nutraceut. Res..

[44] Pragna C.H., Ranjitha Gracy T.K., Mahendran R., Anandharamakrishnan C. (2019). Effects of Microwave and Cold Plasma Assisted Hydrodistillation on Lemon Peel Oil Extraction. Innov. Food Sci. Emerg..

[45] Shi M., Zhang J., Liu C., Cui Y., Li C., Liu Z., Kang W. (2019). Ionic Liquid-Based Ultrasonic-Assisted Extraction to Analyze Seven Compounds in *Psoralea Fructus* Coupled with HPLC. Molecules.

[46] Sun Y., Zhang M., Fang Z. (2020). Efficient physical extraction of active constituents from edible fungi and their potential bioactivities: A review. Trends Food Sci. Tech..

[47] Wen Q., Zhang H., Zhang P., Jiang X. (2005). Improved Artificial Neural Network for Data Analysis and Property Prediction in Slag Glass-Ceramic. J. Am. Ceram. Soc..

[48] Yin L., Deng P., He P., Liu Y., Li L. (2021). Optimization of Total Flavonoid Extraction from *Lilium brownii* Based on Genetic Algorithm-Neural Network and Response Surface Methodology. Food Res. Dev..

