# Supplementary material for: Identification and Optimization of a Novel Taxanes Extraction Process from Taxus cuspidata Needles by High-Intensity Pulsed Electric Field
Source: Molecules. 2022 May 7;27(9):3010. doi: 10.3390/molecules27093010 (PMC9104932; doi:10.3390/molecules27093010)
Supplement: Supplementary file 1 [file molecules-27-03010-s001.zip › molecules-1649645-supplementary.pdf]

Identification and Optimization of a Novel Taxanes Extraction Process from *Taxus cuspidata* Needles by High-Intensity Pulsed Electric Field

Zirui Zhao, Yajing Zhang, Huiwen Meng, Wenlong Li and Shujie Wang *

College of Biology and Agricultural Engineering, Jilin University, Changchun 130022, China; zzirui94@163.com (Z.Z.); yajing20@mails.jlu.edu.cn (Y.Z.); 13834838037@163.com (H.M.); liwl21@mails.jlu.edu.cn (W.L.)

* Correspondence: wangsj12365@163.com

Supplementary File

Supplementary Table S1

Table S1. ANOVA and regression analysis of PBD for prediction of significant extraction variables.

Source	Sum of Squares	Degree of Freedom	Mean Square	F-Value	*p*-Value	Inference
Model	24436.96	7	3490.99	20.40	0.0056	Significance
Residua	684.49	4	171.12			
Cor total	25121.45	11				
$R^2 = 0.9728$; $Radj^2 = 0.9251$						

Term	Mean Square	F-Value	*p*-Value	
A	17105.28	99.96	0.0006	Significance
B	15.46	0.090	0.7787	
C	94.30	0.55	0.4991	
D	5351.81	31.27	0.0050	Significance
E	58.61	0.34	0.5898	
F	3.39	0.020	0.8948	
G	1808.11	10.57	0.0314	Significance

A: electric field strength (kV/cm), *B*: extraction times (time), *C*: solid-liquid ratio (g/mL), *D*: pulse number, *E*: centrifugal speed (r/min), *F*: flow rate (mL/min), *G*: particle size (mesh), TEF: taxanes equivalents of *Taxus cuspidate* (μg/g), R^2: Coefficient of determination, $Radj^2$: adjusted determination coefficients.

Supplementary Table S2

Table S2. ANOVA for response surface polynomial model.

Source	SUN of SQUARES	df	Mean Square	F Value	*p*-Value	Significant
Model	171000	9	18998.49	45.87	<0.0001	***
X_1	59555.63	1	59555.63	143.81	<0.0001	***
X_2	18069.01	1	18069.01	43.63	0.0003	***
X_3	5880.13	1	5880.13	14.20	0.0070	**
X_1X_2	5600.28	1	5600.28	13.52	0.0079	**

X_1X_3	176.89	1	176.89	0.43	0.5343	
X_2X_3	11017.65	1	11017.65	26.60	0.0013	**
X_1^2	28108.93	1	28108.93	67.87	<0.0001	***
X_2^2	11648.85	1	11648.85	28.13	0.0011	**
X_3^2	23782.84	1	23782.84	57.43	0.0001	***
Residual	2898.98	7	414.14			
Lack of Fit	1542.38	3	514.13	1.52	0.3395	
Pure Error	1356.61	4	339.15			
Cor Total	173900	16				
$R^2 = 0.9833$; $Radj^2 = 0.9619$; $Rpred^2 = 0.8459$; Adeq Precision = 19.575						

Level of significance: *** $p < 0.001$, ** $p < 0.01$

X_1: Electric field strength (kV/cm); X_2: Pulse number; X_3: Particle size (mesh).

Supplementary Table S3

Table S3. Virtual samples.

	Electric Field Strength (kV/cm)	Pulse Number	Particle Size (Mesh)
1	15.03	10.02	120.24
2	15.03	10.02	119.76
3	15.03	9.98	120.24
4	15.03	9.98	119.76
5	14.97	10.02	120.24
6	14.97	10.02	119.76
7	14.97	9.98	120.24
8	14.97	9.98	119.76

Supplementary Figure S1

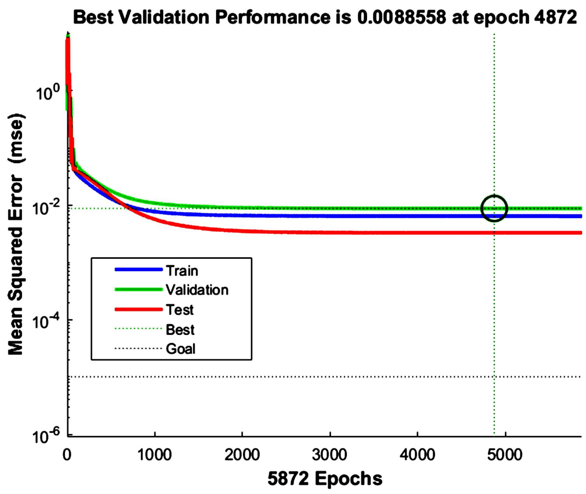

Figure S1. The performance of the ANN model.

Supplementary Figure S2

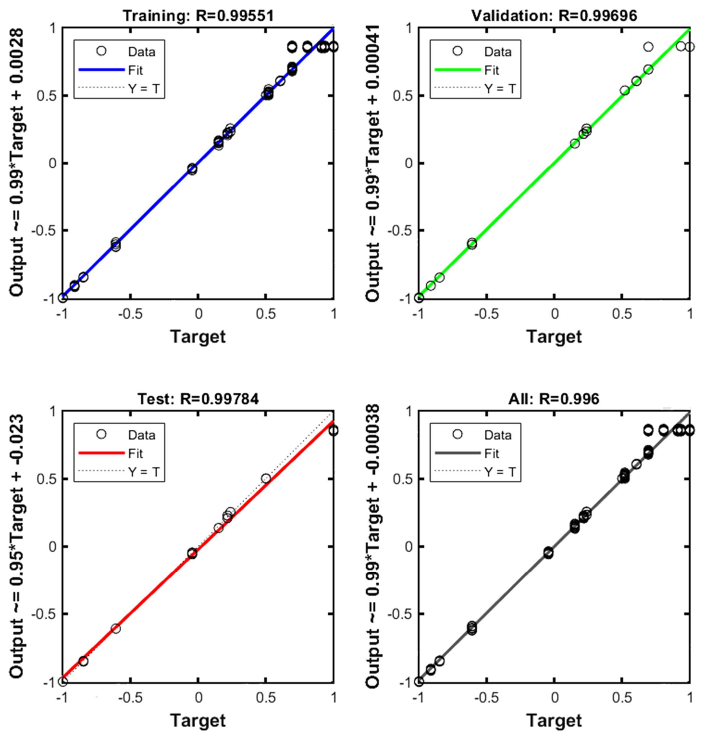

Figure S2. Regression plot of the ANN model for training, testing, validation, and all data sets.

Supplementary Table S4

Table S4. Evaluation of the predictive capacities of RSM and GA-BP modeling systems.

	**RSM**	**GA-BP**
**R^2**	0.9833	0.9921
**RMSE**	13.0301	8.943
**MAPE**	2.10837	0.70595
**SEP**	0.02353	0.01621

RMSE: root mean square error, MAPE: mean absolute percent error, R^2: coefficient of determination, SEP: standard error of prediction, RSM: response surface methodology, GA-BP: back-propagation neural network conjugated with genetic algorithm.

Supplementary Figure S3

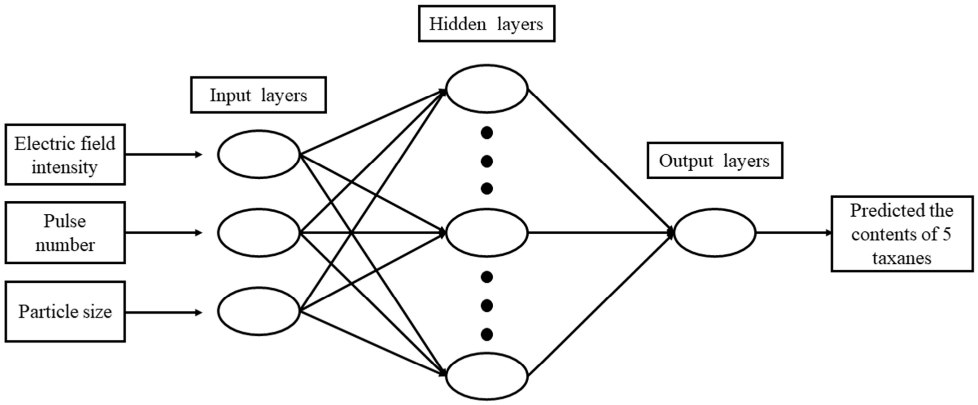

Figure S3. ANN structure of PEF extraction of five main taxanes from *Taxus cuspidate*.
